# The Implicit Association of High-Fat Food and Shame Among Women Recovered From Eating Disorders

**DOI:** 10.3389/fpsyg.2020.01068

**Published:** 2020-06-03

**Authors:** Roni Elran-Barak, Tzipi Dror, Andrea B. Goldschmidt, Bethany A. Teachman

**Affiliations:** ^1^ School of Public Health, University of Haifa, Haifa, Israel; ^2^The School of Psychological Sciences, Tel Aviv University, Tel Aviv, Israel; ^3^Department of Psychiatry and Human Behavior, The Miriam Hospital, Warren Alpert Medical School of Brown University, Providence, RI, United States; ^4^School of Medicine, University of Virginia, Charlottesville, VA, United States

**Keywords:** eating disorders, implicit association, recovery, EAT-26, high-fat food

## Abstract

**Background:**

Despite the growing literature about recovery from eating disorders (EDs), it is still unknown if women who report being recovered from EDs present with differing implicit attitudes about high-fat (vs. low-fat) food relative to women who report having a current ED and women who report never having had an ED.

**Methods:**

Female volunteers (*N* = 2,785) to the Project Implicit Mental Health (PIMH) website (https://implicit.harvard.edu/) were divided into three ED groups: current ED (*n* = 335), prior ED (*n* = 393), and healthy controls (*n* = 1,843). Participants completed, between 2016 and 2017, a background questionnaire, together with the Implicit Association Test (IAT), measuring implicit associations between high-fat (vs. low-fat) food and shame (vs. acceptableness). Linear regression models were conducted to examine cross-sectional differences between groups.

**Results:**

Women with prior EDs had stronger implicit associations relative to healthy controls (*p* = 0.041) and similar implicit associations relative to women with current EDs (*p* = 0.424).

**Discussion:**

The implicit association between high-fat food and shame may not diminish over time among women with EDs. Future longitudinal studies are warranted to clarify whether an experience of EDs may leave a “scar,” manifested in specific implicit associations, that may potentially lead to recurrence after remission.

## Introduction

Eating disorders (EDs) are complex psychiatric conditions that are often associated with a chronic course and a long recovery process (Wagner et al., 2006; Tomba et al., 2019). Recent longitudinal findings (Eddy et al., 2017) suggest poor long-term prognosis, with one-third to two-thirds of individuals with anorexia nervosa (AN) and bulimia nervosa (BN) still struggling with an ED after 9 years of follow-up. Many recovered individuals continue to struggle with various psychological/psychiatric conditions (Wagner et al., 2006) that persist for many years after recovery (Bardone-Cone et al., 2010), and many characteristics that are prevalent among currently ill patients (e.g., negative body image) (Eshkevari et al., 2014) improve only slightly after recovery, while remaining somewhat impaired relative to healthy controls (Yackobovitch-Gavan et al., 2009; Bardone-Cone et al., 2010). Nevertheless, studies examining implicit associations toward high-fat food among those recovered from EDs are scarce.

Implicit associations refer to representations in memory that link a stimulus and an involuntarily activated evaluative outcome, which do not require conscious reflection to influence affect, cognition, or behavior (Teachman et al., 2019). Measures of implicit associations, and primarily the Implicit Association Test (IAT) (Greenwald et al., 1998), are relatively insensitive to social desirability pressures or to other potential response biases (Nosek, 2005), and they often add explanatory power, sometimes even surpassing the predictive value of explicit self-report measures (Hofmann et al., 2005; Greenwald et al., 2009). Moreover, implicit associations between concepts can help understand the formation and expression of thoughts, attitudes, and actions (Vartanian et al., 2005). For example, implicit associations show small to moderate positive relationships with self-reported mental health symptoms (Werntz et al., 2016) while adding to the prediction of these symptoms above and beyond explicit associations, pointing to the potential future clinical utility and validity of implicit associations.

Few studies have used implicit measures in the study of EDs. There is evidence that patients with AN display significantly greater implicit negative attitudes toward high-calorie food relative to recovered patients and healthy controls (Spring and Bulik, 2014). Additionally, Werntz et al. (2016), who used the IAT among a large convenience sample (using a subset of the data we analyzed for the present study), suggested that the implicit associations of high-fat food with shame may be linked with ED symptoms. Potentially contradictory findings (Neimeijer et al., 2015; Paslakis et al., 2016) suggest that food-related implicit approach/avoidance tendencies do not necessarily play an important role in EDs: No difference was found between patients with AN and healthy controls in the effect of caloric content of food on implicit tendencies. Relatedly, a study using the IAT (Roefs and Jansen, 2002) reports that relative to people with normal weight, people with obesity may have a significantly stronger implicit negative attitude toward high-fat foods, which may be contradictory to their preferences and behaviors.

Despite the growing literature about recovery from EDs and despite the understanding that it is important to characterize women who have recovered from EDs (e.g., in order to tailor interventions to reduce rates of relapse), it is unknown if women recovered from ED present with differing implicit attitudes about high-fat food relative to currently ill patients and healthy controls. A better understanding of the implicit associations of women recovered from EDs would help identify whether an experience of an ED leaves a “scar” that is manifested in specific implicit associations and may potentially lead to recurrence after remission (van Tuijl et al., 2016).

We examined the differential association of the two target categories (high-fat vs. low-fat food) with the attribute dimension (shameful vs. acceptable) among a large convenience sample of women who report having a current ED, being recovered from EDs, or never having had an ED. The following research question was identified:

Does the implicit association of high-fat food and shame differ between women with current EDs, women with prior EDs, and healthy controls?

Based on the extant recovery literature (Wagner et al., 2006; Tomba et al., 2019), we hypothesized that relative to women with prior EDs, women with current EDs would have *stronger*, and healthy controls would have *weaker*, implicit associations of high-fat food and shame. ED symptoms and the explicit association of high-fat food and shame were also assessed in order to shed light on the relationships between implicit and explicit measures.

## Materials and Methods

### Procedure

Data were retrieved from the Project Implicit Mental Health (PIMH) website (Glenn et al., 2017), a demonstration and research public website, and a sister site to the Project Implicit website^1^ (Nosek, 2005), which allows visitors to learn about their implicit biases. Volunteers to the PIMH website self-selected a mental health domain (alcohol, anxiety, depression, or eating) and completed the informed consent form. Only volunteers who self-selected to participate in the eating IAT study between May 2016 and September 2017 were included. Although volunteers were not specifically asked to select a domain that was most relevant to them, it could potentially be that domain selection was related to personal preferences. Following consent, participants completed, in random order, the questionnaire and the IAT (Greenwald et al., 1998). An opportunity to view the IAT score and receive feedback about implicit attitudes was then provided.

### Measures

#### Eating Disorder History

Volunteers were prompted with the introductory sentence: *“The next questions ask for insight into your current and past mental health challenges.*” Volunteers who opted to proceed were asked: “*Are you currently, or have you ever struggled with moderate to severe mental or emotional difficulties (e.g., alcohol or drug problems, depression, panic attacks, anxiety, eating disorder, attention deficit, etc.) that lasted a minimum of several weeks and interfered with your daily life? [yes/no].”* Volunteers who selected the “yes” response were then asked: *“We are interested in your current and past mental health difficulties. Please read the following list of disorders and indicate whether you are currently struggling or have struggled in the past with these types of mental illnesses. If you have never struggled with any of the disorders below, please leave this form blank and click Continue.”* A list of disorders was then provided. Participants who checked that they were currently struggling with EDs were classified as “current ED,” participants who checked that they have struggled with EDs in the past were classified as “prior EDs,” and the rest were classified as “never ED” or healthy controls. A total of 106 participants who reported that their ED was both current and past were classified as currently ill.

#### Eating Attitudes Test

Eating disorder symptoms were assessed based on the 26 items of the Eating Attitudes Test (EAT-26) (Garner and Garfinkel, 1979), one of the most common questionnaires in the ED literature. The total score was computed by summing all 26 items (Garner and Garfinkel, 1979), and separate scores were computed for three subscales: Dieting, Bulimia, and Oral Control. Higher scores reflect more severe ED symptoms. A score at or above 20 on the EAT-26 indicates a high level of concern about dieting, body weight, or problematic eating behaviors.

#### Explicit Association Measure

Participants answered two questions: (1) *To what extent do you think of low-fat foods as acceptable?* (2) *To what extent do you think of high-fat foods as shameful?* Possible responses were (coded 1–9): extremely acceptable to extremely shameful. We computed the explicit association measure by subtracting participants’ response to the “low-fat” question from their response to the “high-fat” question (Werntz et al., 2016). The difference score was computed to create a relative explicit association measure that would match the relative structure of the implicit measure.

#### Implicit Association Test (Greenwald et al., 1998)

The four category labels in the current IAT were *high-fat food, low-fat food, shameful*, and *acceptable*. The stimuli for the food categories were words describing high-fat foods (*fries, cake, candy, chocolate*) and low-fat foods (*salad, carrots, fruit, cucumber*). The stimuli for the attribute categories were words that were related to the concepts “shameful” (*disgraceful, bad, embarrassing, shameful*) and “acceptable” (*suitable, good, appropriate, acceptable*). In the IAT, participants categorize the stimuli using two computer keys. The categories appear on the upper left and upper right corners of the screen. In the critical blocks, participants respond with one key to the stimuli of two categories (e.g., “high-fat food” and “shameful”) and respond with the other key to the stimuli that belong to the other two categories (e.g., “low-fat food” and “acceptable”). The IAT consisted of seven blocks and followed the standard procedure described by Nosek et al. (2005). The IAT D score was computed according to the algorithm recommended by Greenwald et al. (2003) such that a higher (positive) score reflected a stronger implicit association between the concepts “high-fat food” (vs. “low-fat food”) and “shameful” (vs. “acceptable”). Large positive IAT D scores suggest greater associative strength for the hypothesized congruent pairings, indicative of a stronger associative strength for high-fat (vs. low-fat) food and shameful (vs. acceptable). Large negative IAT D scores indicate implicit preference in the reverse direction of that hypothesized. IAT D measures at or near zero indicate little to no implicit evaluative preference for either end of the bipolar target concept under investigation. Following the suggested effect size guidelines used by Greenwald et al. (Nosek et al., 2002), IAT D of 0.15–0.35 is slight, 0.35–0.65 is moderate, and greater than 0.65 is a strong effect. The task is interpreted in relative terms, but we will refer to it as the implicit association between high-fat food and shame throughout the manuscript for ease of reference.

### Statistical Analyses

Analyses were conducted in SPSS-23. Participants (*N* = 2,785 women) were divided into two ED groups based on self-report: current ED (*n* = 335) and prior ED (*n* = 393). Participants who reported that they didn’t suffer from current or past EDs were classified as healthy controls (*n* = 2,057). Male participants were omitted from final analyses due to negligible participation rate among the two ED groups. In order to maintain reliability between self-report measures, females in the never-ED group who had a score of above 20 on the EAT-26 questionnaire (*n* = 254) were omitted from the analyses. Hence the healthy control group consisted of 1,843 women. ANOVA with *post hoc* comparisons was used to examine differences in the independent variables (ED symptoms and the implicit/explicit association of high-fat food and “shameful”) among the three groups. Linear regressions were fitted to examine differences between the groups (i.e., prior vs. current ED, and prior vs. never ED) in the independent variables while adjusting for age, BMI, and education.

## Results

Participants were mostly White (64.9% White, 9.1% Asian, 12.5% Hispanic, 6.6% African American), residing in 72 different countries (64.3% from the United States). As depicted in Table 1, participants were, on average, 27.6 (10.92) years old, with an average BMI of 25.2 (8.22) kg/m^2^.

**TABLE 1:** Characteristics of female participants by eating disorder (ED) history.

	**Current ED*n* = 335**	**Prior ED*n* = 393**	**Healthy Controls*n* = 1,843**	**Entire Sample*N* = 2,571**
**Background variables**				
Age (*SD*)	24.98 (9.21)	26.36 (8.79)	28.30 (11.51)	27.57 (10.92)
BMI (*SD*)	26.00 (8.92)	25.16 (12.19)	25.06 (6.89)	25.20 (8.22)
High education *n* (%)	271 (81.9)	326 (84.2)	1682 (84.0)	2279 (83.8)
**Dependent variables**				
ED symptoms^1^ (*SD*)	26.51 (14.14)	15.97 (11.35)	6.75 (5.18)	10.73 (10.63)
Explicit associations^2^ (*SD*)	3.35 (2.78)	2.41 (2.81)	1.67 (2.32)	2.00 (2.53)
Implicit associations^3^ (*SD*)	0.73 (0.41)	0.72 (0.43)	0.68 (0.45)	0.69 (0.45)

*^1^Eating Attitudes Test (EAT-26). ^2^The explicit associations of high-fat food and shame. ^3^The Implicit Association Test (IAT) measuring associations of high-fat food and shame.*

The mean EAT-26 score was 10.73 (10.6), and mean implicit/explicit scores were 0.69 (0.45) and 2.00 (2.53), respectively. We conducted one-way ANOVA with *post hoc* comparisons to examine differences in the independent variables among the three groups. There were significant group differences in terms of ED symptoms [*F*(2,2568) = 950.5, *p* < 0.001] and in terms of the explicit association [*F*(2,2532) = 72.0, *p* < 0.001], such that women with current EDs had significantly higher scores than women with prior EDs (*p* < 0.001), and women with prior EDs had higher scores than healthy controls (*p* < 0.001). In contrast, there were no significant group differences in terms of the implicit association [*F*(2,2435) = 2.5, *p* = 0.085].

Correlations between the IAT score and the other dependent explicit variables were examined as follows: The IAT score was weakly (but reliably) related to the explicit belief that high-fat food is shameful (*r* = 0.150, *p* < 0.001), to the total EAT-26 score (*r* = 0.094, *p* < 0.001), and to one out of the three subscales of the EAT-26 (Dieting: *r* = 0.117, *p* < 0.001; Bulimia: *r* = 0.032, *p* = 0.113; and Oral Control: *r* = 0.022, *p* = 0.276). In addition, the following three EAT-26 items had the strongest correlations with the IAT score: *“I am terrified about being overweight,” “I particularly avoid food with a high carbohydrate content,” and “I avoid foods with sugar in them.”*

We examined group differences while adjusting for background variables (age, education, BMI). Table 2 reports three linear regression models to predict ED symptoms, the explicit associations, and the implicit associations. Relative to women in the prior-ED group, women in the never-ED group were *less* likely to have a higher EAT-26 score (β = −0.38, *p* < 0.001) and *less* likely to have stronger explicit (β = −0.14, *p* < 0.001) and implicit associations (β = −0.05, *p* = 0.041). Nevertheless, relative to women in the prior-ED group, women in the current-ED group were *more* likely to a have higher EAT-26 score (β = 0.35, *p* < 0.001) and *more* likely to have stronger explicit (β = 0.11, *p* < 0.001) but not implicit (β = 0.02, *p* = 0.424) associations. In other words, our hypothesis was rejected, as relative to women with current EDs, women with prior EDs had a similar (and not weaker) implicit association between the concepts “high-fat food” (vs. “low-fat food”) and “shameful” (vs. “acceptable”). The same regression analyses were conducted again only among United States residents, with similar significant results.

**TABLE 2:** Linear regression models to predict ED symptoms and the implicit/explicit associations of high-fat food and shame among female participants (*N* = 2,571).

	**ED Symptoms**^1^			**Explicit Associations**^2^			**Implicit Associations**^3^		
			
	**Standardized Beta**	**Confidence Interval 95% CI**	***P*-value**	**Standardized Beta**	**Confidence Interval 95% CI**	***P*-value**	**Standardized Beta**	**Confidence Interval 95% CI**	***P*-value**
Age	–0.074	[−0.109, −0.041]	<0.001	–0.141	[−0.186, −0.099]	<0.001	0.185	[0.142, 0.230]	<0.001
BMI	–0.029	[−0.070, 0.004]	0.077	0.081	[0.045, 0.139]	<0.001	–0.004	[−0.052, 0.043]	0.843
High education	–0.014	[−0.047, 0.018]	0.376	0.009	[−0.033, 0.051]	0.667	0.017	[−0.025, 0.060]	0.427
Current vs. prior ED	0.354	[0.312, 0.389]	<0.001	0.105	[0.053, 0.152]	<0.001	0.021	[−0.030, 0.071]	0.424
Never ED vs. prior ED	–0.375	[−0.414, −0.336]	<0.001	–0.136	[−0.185, −0.084]	<0.001	–0.054	[−0.104, −0.002]	0.041

*^1^EAT-26. ^2^The explicit associations of high-fat food and shame. ^3^The IAT measuring associations of high-fat food and shame.*

## Discussion

Data indicated that women with prior EDs had *stronger* implicit associations of high-fat food and shame relative to healthy controls (though the effect was small) and *similar* implicit associations of high-fat food and shame relative to women with current EDs. This finding may provide an interesting addition to the recovery literature (Wagner et al., 2006) by suggesting that negative implicit attitudes toward high-fat food may not reliably diminish over time.

Previous studies suggest that many characteristics that are prevalent among currently ill patients (e.g., negative body image) improve significantly after recovery, while remaining slightly impaired relative to healthy control participants (Oldershaw et al., 2010; Eshkevari et al., 2014). In contrast, our data suggested that women with current and past EDs do not differ significantly in their implicit associations between high-fat food and shame. This discrepancy between studies might be due to the fact that our study used implicit measures (which are relatively insensitive to social desirability pressures or to other potential response biases), while other studies relied on explicit measures. Furthermore, this present study used a large non-clinical sample, while prior studies relied mainly on clinical samples. It is important to use non-clinical samples, as it is well documented (Swanson and Field, 2016; Forrest et al., 2017) that clinical and non-clinical samples have different characteristics, including different recovery patterns (e.g., there is a slight proportion of people who don’t seek treatment and recover spontaneously) (Vandereycken, 2012). Moreover, as a result of Berkson’s bias (Berkson, 1946), individuals with more severe symptoms are more likely to seek clinical care, thereby artificially inflating illness severity in clinical samples.

The World Health Organization’s nutritional guidelines (WHO, 2018) recommend limiting the consumption of the target high-fat foods (e.g., cakes, chocolates, candies, fries) and increasing the consumption of the target low-fat foods (e.g., vegetables, fruits). Therefore, it is reasonable to suggest that the implicit association between high-fat foods and shame-related words (e.g., bad, disgraceful, embarrassing, shameful) reflects an acceptance, and perhaps an internalization, of nutritional guidelines, regardless of ED status. Moreover, it could be that some of our female participants, specifically those with past or current EDs, were simply more aware of these nutritional guidelines due to their preoccupation with food-related issues. Future longitudinal studies are warranted to clarify whether the examined implicit associations reflect a stronger acceptance of nutritional guidelines or, alternatively, a “scar” that may potentially lead to a recurrence after remission.

In light of nutritional guidelines (WHO, 2018) public health professionals may view a stronger implicit negative association with high-fat foods as desirable. There are contrasting views from different professional camps on the best prevention and treatment strategies for obesity and EDs and for those individuals with both obesity and EDs (i.e., how to promote limiting the consumption of high-fat food while not promoting disordered eating) (Nuemark-Sztainer, 2003). In addition, given that not all ED treatments target implicit beliefs about high-fat food, it could be that we can’t expect changes in these associations after recovery. Therefore, a better understanding of the impact that these implicit associations may have on rates of relapse will inform ED treatment and prevention programs.

Strengths of the current study include the recruitment of a large non-clinical sample and the use of implicit measures that are relatively insensitive to social desirability pressures or to other potential response biases. Several limitations should be noted. *First*, although the current sample was very large and heterogeneous, it was not a representative sample. Participants voluntarily selected to take the IAT online. Nevertheless, data collected from Project Implicit have been studied intensively for several years, and the validity of results is comparable to that of similar data collected in experimental laboratory conditions (Nosek et al., 2002; Klein et al., 2014). *Second*, the value in using the IAT to measure implicit social cognition has been criticized by some researchers (Blanton et al., 2009). *Third*, height and weight, as well as past and present ED diagnoses, were assessed by self-report, which is likely less accurate than objective measurement and clinical records. However, studies show that web responders usually provide accurate information about themselves (Kraut et al., 2004). Relatedly, we do not have information about participants’ specific ED diagnosis, and it may be that implicit attitudes differ among women with AN, BN, and binge-eating disorder. *Lastly*, it is important to remember that clinicians and researchers are not in agreement about the definition of recovery from EDs (e.g., partial vs. full abstinence from symptoms) (Slof-Op’t Landt et al., 2019), and it could be that a few participants perceive themselves to have had prior EDs, although they still cope with a few ED symptoms.

To conclude, the present study examined implicit associations about high-fat food among women with current and prior EDs. Findings suggested that the implicit belief that high-fat food is shameful may not diminish among women with prior EDs (relative to women with current EDs). Future studies are warranted to confirm our cross-sectional findings via the use of prospective designs with clinical assessments of EDs (as opposed to self-report). Prospective designs are warranted, as a causal inference is essential for the complete understanding of the relations between recovery from EDs and implicit associations.

## Data Availability Statement

The datasets generated for this study are available on request to the corresponding author.

## Ethics Statement

The studies involving human participants were reviewed and approved by the University of Virginia Institutional Review Board for the Social and Behavioral Sciences. The patients/participants provided their written informed consent to participate in this study.

## Author Contributions

RE-B initiated the idea and wrote the first draft. BT collected the data. RE-B and TD performed the computations. All authors verified the analytical methods, discussed the results, and contributed to the final manuscript.

## Conflict of Interest

The authors declare that the research was conducted in the absence of any commercial or financial relationships that could be construed as a potential conflict of interest.

## Footnotes

1 https://implicit.harvard.edu/

## References

[B1] Bardone-Cone A. M. Harney M. B. Maldonado C. R. Lawson M. A. Robinson D. P. Smith R. (2010). Defining recovery from an eating disorder: conceptualization, validation, and examination of psychosocial functioning and psychiatric comorbidity. *Behav. Res. Ther.* 48 194–202. 10.1016/j.brat.2009.11.001 19945094 PMC2829357

[B2] Berkson J. (1946). Limitations of the application of fourfold table analysis to hospital data. *Biometr. Bull.* 2 47–53. 21001024

[B3] Blanton H. Jaccard J. Klick J. Mellers B. Mitchell G. Tetlock P. E. (2009). Strong claims and weak evidence: reassessing the predictive validity of the IAT. *J. Appl. Psychol.* 94 567–582. 10.1037/a0014665 19449998

[B4] Eddy K. T. Tabri N. Thomas J. J. Murray H. B. Keshaviah A. Hastings E. (2017). Recovery from anorexia nervosa and bulimia nervosa at 22-year follow-up. *J. Clin. Psychiatry* 78 184–189. 10.4088/JCP.15m10393 28002660 PMC7883487

[B5] Eshkevari E. Rieger E. Longo M. R. Haggard P. Treasure J. (2014). Persistent body image disturbance following recovery from eating disorders. *Int. J. Eat. Disord.* 47 400–409. 10.1002/eat.22219 24243423

[B6] Forrest L. N. Smith A. R. Swanson S. A. (2017). Characteristics of seeking treatment among US adolescents with eating disorders. *Int. J. Eat. Disord.* 50 826–833. 10.1002/eat.22702 28323350

[B7] Garner D. M. Garfinkel P. E. (1979). The eating attitudes test: an index of the symptoms of anorexia nervosa. *Psychol. Med.* 9 273–279. 10.1017/s0033291700030762 472072

[B8] Glenn J. J. Werntz A. J. Slama S. J. Steinman S. A. Teachman B. A. Nock M. K. (2017). Suicide and self-injury-related implicit cognition: a large-scale examination and replication. *J. Abnorm. Psychol.* 126 199–211. 10.1037/abn0000230 27991808 PMC5305619

[B9] Greenwald A. G. McGhee D. E. Schwartz J. L. K. (1998). Measuring individual differences in implicit cognition: the implicit association test. *J. Personal. Soc. Psychol.* 74 1464–1480. 10.1037//0022-3514.74.6.1464 9654756

[B10] Greenwald A. G. Nosek B. A. Banaji M. R. (2003). Understanding and using the implicit association test: I. An improved scoring algorithm. *J. Personal. Soc. Psychol.* 85 197–216. 10.1037/0022-3514.85.2.197 12916565

[B11] Greenwald A. G. Poehlman T. A. Uhlmann E. L. Banaji M. R. (2009). Understanding and using the Implicit association test: III. Meta-analysis of predictive validity. *J. Personal. Soc. Psychol.* 97 17–41. 10.1037/a0015575 19586237

[B12] Hofmann W. Gawronski B. Gschwendner T. Le H. Schmitt M. (2005). A meta-analysis on the correlation between the implicit association test and explicit self-report measures. *Personal. Soc. Psychol. Bull.* 31 1369–1385. 10.1177/0146167205275613 16143669

[B13] Klein R. A. Ratliff K. A. Vianello M. Adams R. B. Jr. Bahník Š. Bernstein M. J. (2014). Investigating variation in replicability: a “many labs” replication project. *Soc. Psychol*. 45, 142–152. 10.1027/1864-9335/a000178

[B14] Kraut R. Olson J. Banaji M. Bruckman A. Cohen J. Couper M. (2004). Psychological research online: report of board of scientific affairs’ advisory group on the conduct of research on the internet. *Am. Psychol.* 59 105–117. 10.1037/0003-066x.59.2.105 14992637

[B15] Neimeijer R. A. M. de Jong P. J. Roefs A. (2015). Automatic approach/avoidance tendencies towards food and the course of anorexia nervosa. *Appetite* 91 28–34. 10.1016/j.appet.2015.03.018 25817483

[B16] Nosek B. A. (2005). Moderators of the relationship between implicit and explicit evaluation. *J. Exp. Psychol. Gen.* 134 565–584. 10.1037/0096-3445.134.4.565 16316292 PMC1440676

[B17] Nosek B. A. Banaji M. R. Greenwald A. G. (2002). Harvesting implicit group attitudes and beliefs from a demonstration web site. *Group Dyn. Theory Res. Pract.* 6 101–115. 10.1037/1089-2699.6.1.101

[B18] Nosek B. A. Greenwald A. G. Banaji M. R. (2005). Understanding and using the Implicit association test: II. Method variables and construct validity. *Personal. Soc. Psychol. Bull.* 31 166–180. 10.1177/0146167204271418 15619590

[B19] Nuemark-Sztainer D. (2003). Obesity and eating disorder prevention: an integrated approach? *Adolesc. Med. Clin.* 14 159–173. 12529199

[B20] Oldershaw A. Hambrook D. Tchanturia K. Treasure J. Schmidt U. (2010). Emotional theory of mind and emotional awareness in recovered anorexia nervosa patients. *Psychos. Med.* 72 73–79. 10.1097/PSY.0b013e3181c6c7ca 19995886

[B21] Paslakis G. Kühn S. Schaubschläger A. Schieber K. Röder K. Rauh E. (2016). Explicit and implicit approach vs. avoidance tendencies towards high vs. low calorie food cues in patients with anorexia nervosa and healthy controls. *Appetite* 107 171–179. 10.1016/j.appet.2016.08.001 27496787

[B22] Roefs A. Jansen A. (2002). Implicit and explicit attitudes toward high-fat foods in obesity. *J. Abnorm. Psychol.* 111 517–521. 10.1037/0021-843x.111.3.517 12150428

[B23] Slof-Op’t Landt M. C. T. Dingemans A. E. de la Torre Y. Rivas J. van Furth E. F. (2019). Self-assessment of eating disorder recovery: absence of eating disorder psychopathology is not essential. *Int. J. Eat. Disord.* 52 956–961. 10.1002/eat.23091 31058337

[B24] Spring V. L. Bulik C. M. (2014). Implicit and explicit affect toward food and weight stimuli in anorexia nervosa. *Eat. Behav.* 15 91–94. 10.1016/j.eatbeh.2013.10.017 24411758

[B25] Swanson S. A. Field A. E. (2016). Commentary: considerations for the use of registry data to study adolescent eating disorders. *Int. J. Epidemiol.* 45 488–490. 10.1093/ije/dyw084 27097750

[B26] Teachman B. A. Clerkin E. M. Cunningham W. A. Dreyer-Oren S. Werntz A. (2019). Implicit cognition and psychopathology: looking back and looking forward. *Annu. Rev. Clin. Psychol.* 15 123–148. 10.1146/annurev-clinpsy-050718-095718 30633549

[B27] Tomba E. Tecuta L. Crocetti E. Squarcio F. Tomei G. (2019). Residual eating disorder symptoms and clinical features in remitted and recovered eating disorder patients: a systematic review with meta-analysis. *Int. J. Eat. Disord.* 52 759–776. 10.1002/eat.23095 31169332

[B28] van Tuijl L. A. Glashouwer K. A. Bockting C. L. H. Tendeiro J. N. Penninx B. W. J. H. de Jong P. J. (2016). Implicit and explicit self-esteem in current, remitted, recovered, and comorbid depression and anxiety disorders: the NESDA study. *PLoS One* 11:e0166116. 10.1371/journal.pone.0166116 27846292 PMC5112909

[B29] Vandereycken W. (2012). Self-change in eating disorders: is “spontaneous recovery” possible? *Eat. Disord.* 20 87–98. 10.1080/10640266.2012.653942 22364341

[B30] Vartanian L. R. Peter Herman C. Polivy J. (2005). Implicit and explicit attitudes toward fatness and thinness: the role of the internalization of societal standards. *Body Image* 2 373–381. 10.1016/j.bodyim.2005.08.002 18089202

[B31] Wagner A. Barbarich-Marsteller N. C. Frank G. K. Bailer U. F. Wonderlich S. A. Crosby R. D. (2006). Personality traits after recovery from eating disorders: do subtypes differ? *Int. J. Eat. Disord.* 39 276–284. 10.1002/eat.20251 16528697

[B32] Werntz A. J. Steinman S. A. Glenn J. J. Nock M. K. Teachman B. A. (2016). Characterizing implicit mental health associations across clinical domains. *J. Behav. Ther. Exp. Psychiatry* 52 17–28. 10.1016/j.jbtep.2016.02.004 26962979 PMC4871740

[B33] WHO, (2018). *Healthy diet.* Avaliable at: https://www.who.int/news-room/fact-sheets/detail/healthy-diet (accessed April 27, 2020).

[B34] Yackobovitch-Gavan M. Golan M. Valevski A. Kreitler S. Bachar E. Lieblich A. (2009). An integrative quantitative model of factors influencing the course of anorexia nervosa over time. *Int. J. Eat. Disord.* 42 306–317. 10.1002/eat.20624 19040269

